# OvHV-2 Glycoprotein B Delivered by a Recombinant BoHV-4 Is Immunogenic and Induces Partial Protection against Sheep-Associated Malignant Catarrhal Fever in a Rabbit Model

**DOI:** 10.3390/vaccines9020090

**Published:** 2021-01-26

**Authors:** Smriti Shringi, Donal O’Toole, Emily Cole, Katherine N. Baker, Stephen N. White, Gaetano Donofrio, Hong Li, Cristina W. Cunha

**Affiliations:** 1 Department of Veterinary Microbiology and Pathology, Washington State University, Pullman, WA 99164, USA; smriti_shringi@wsu.edu (S.S.); emily.cole@wsu.edu (E.C.); kayak@wsu.edu (K.N.B.); stephen.white@usda.gov (S.N.W.); 2 Department of Veterinary Sciences, University of Wyoming, Laramie, WY 82070, USA; DOT@uwyo.edu; 3 Animal Disease Research Unit, Agricultural Research Service, USDA, Pullman, WA 99164, USA; hlidyz@gmail.com; 4 Center for Reproductive Biology, Washington State University, Pullman, WA 99164, USA; 5 Department of Medical-Veterinary Science, University of Parma, 43126 Parma, Italy; gaetano.donofrio@unipr.it; 6 Paul G. Allen School for Global Animal Health, Washington State University, Pullman, WA 99164, USA

**Keywords:** viral-vectored vaccine, bovine herpesvirus-4, ovine herpesvirus-2, gammaherpesvirus, malignant catarrhal fever, rabbit model, glycoprotein B

## Abstract

An efficacious vaccine for sheep-associated malignant catarrhal fever (SA-MCF) is important for the livestock industry. Research towards SA-MCF vaccine development is hindered by the absence of culture systems to propagate the causative agent, ovine herpesvirus-2 (OvHV-2), which means its genome cannot be experimentally modified to generate an attenuated vaccine strain. Alternative approaches for vaccine development are needed to deliver OvHV-2 antigens. Bovine herpesvirus 4 (BoHV-4) has been evaluated as a vaccine vector for several viral antigens with promising results. In this study, we genetically engineered BoHV-4 to express OvHV-2 glycoprotein B (gB) and evaluated its efficacy as an SA-MCF vaccine using a rabbit model. The construction of a viable recombinant virus (BoHV-4-AΔTK-OvHV-2-gB) and confirmation of OvHV-2 gB expression were performed in vitro. The immunization of rabbits with BoHV-4-AΔTK-OvHV-2-gB elicited strong humoral responses to OvHV-2 gB, including neutralizing antibodies. Following intra-nasal challenge with a lethal dose of OvHV-2, 42.9% of the OvHV-2 gB vaccinated rabbits were protected against SA-MCF, while all rabbits in the mock-vaccinated group succumbed to SA-MCF. Overall, OvHV-2 gB delivered by the recombinant BoHV-4 was immunogenic and partly protective against SA-MCF in rabbits. These are promising results towards an SA-MCF vaccine; however, improvements are needed to increase protection rates.

## 1. Introduction

Ovine herpesvirus 2 (OvHV-2) causes a frequently fatal disease called sheep-associated malignant catarrhal fever (SA-MCF) in several ungulates, such as bison, cattle, pigs, and deer [1,2]. In the absence of effective treatments for the disease, vaccine development comes as an option for the livestock industry. SA-MCF can have significant economic impact, particularly in the growing bison industry in the US and Canada, due to the high susceptibility of bison to the disease [3]. Although cattle are more resistant to SA-MCF than bison, the disease remains an intermittent problem for the cattle industry as losses can on occasion be high [4].

One of the major constraints for the development of an SA-MCF vaccine is the inability to propagate OvHV-2 in cell culture. Since the virus cannot be modified or attenuated in vitro, alternative approaches for delivering OvHV-2 antigens for immunization are of utmost importance.

Similar to all herpesviruses, glycoproteins on the OvHV-2 envelope are essential for initial viral-host interactions that result in viral entry into cells [5]. For instance, we demonstrated that OvHV-2 is unable to infect rabbits after treatment with hyperimmune sera containing antibodies specific for its envelope proteins, including anti-glycoprotein (g) B [6]. This suggests that the antibodies present in the sera blocked the virus, preventing entry into cells and consequent infection. Therefore, gB is considered a promising vaccine candidate to protect end-stage hosts from infection and terminal disease caused by OvHV-2. 

Vector vaccine technology is an attractive way to deliver antigens that has been used successfully in veterinary vaccine development [7]. Bovine herpesvirus-4 (BoHV-4) has been identified as a reliable vaccine delivery vector [8,9,10]. The characteristics of BoHV-4 that favor its use as a vaccine vector include its capability of infecting a variety of hosts with low or no pathogenicity and accommodating large amounts of foreign genetic material. In addition, the BoHV-4 genome is available as a bacterial artificial chromosome (BAC), which tremendously facilitates its manipulation for vaccine purposes [11]. 

Herein, we describe the construction of a recombinant BoHV-4 expressing OvHV-2 gB, BoHV-4-AΔTK-OvHV-2-gB, which was evaluated as an experimental vaccine for SA-MCF. The administration of BoHV-4-AΔTK-OvHV-2-gB in a rabbit model induced a humoral response to OvHV-2 gB that included neutralizing antibodies and conferred partial protection against SA-MCF following challenge with OvHV-2.

## 2. Materials and Methods

### 2.1. Mammalian Cells

Immortalized fetal mouflon sheep kidney cells (FMSK^htert.1^), FMSK^htert.1^ expressing Cre recombinase (FMSK^htert.1^/Cre), and human embryonic kidney (HEK) 293T cells were cultured in complete Dulbecco’s Modified Eagle Medium (c-DMEM glutamax-1 containing 10% FBS, 100 U/mL penicillin, 100 ug/mL streptomycin, and 1 ug/mL amphotericin B) and maintained at 37 °C and 5% CO_2_. FMSK^htert.1^ cells were used for BAC transfections and virus infections, FMSK^htert.1^/Cre cells were used to remove the loxP flanked BAC cassette. HEK293T cells were used for protein expression.

### 2.2. Construction of a BoHV-4 Recombinant Virus 

**Figure 1:**
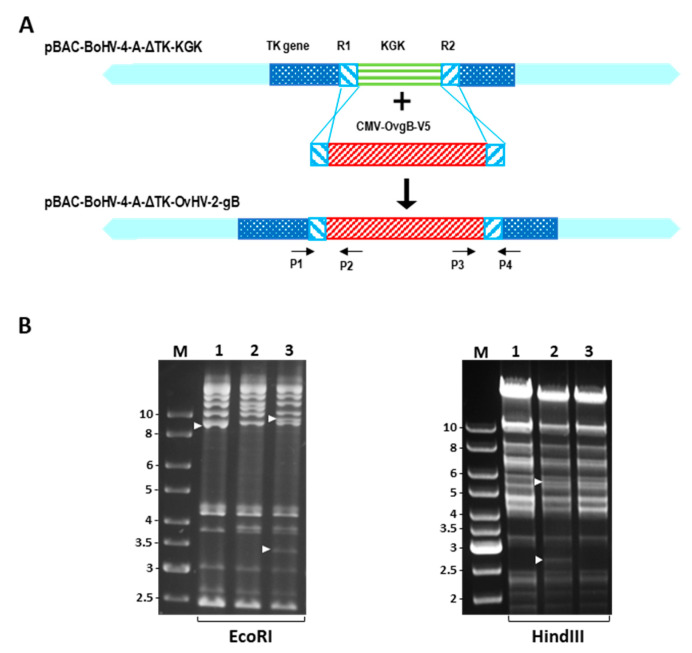
Construction of the recombinant BoHV-4 expressing OvHV-2 gB. (**A**) Diagram illustrates the replacement of the KGK cassette in the TK locus of pBAC-BoHV-4-AΔTK-KGK with the OvHV-2 ORF8 cassette (CMV-OvgB-V5) to generate pBAC-BoHV-4-AΔTK-OvHV-2-gB. Homologous recombination was performed using the R1 and R2 sequences present in the BoHV-4 TK gene using a galactokinase K selection system in *E. coli* SW102. (**B**) Restriction profile following digestion with *Eco*RI and *Hin*dIII showing the integrity of the constructed viruses. Lanes: M, GeneRuler DNA Ladder molecular size marker (in Kbp); 1, pBAC-BoHV-4-A wild-type; 2, pBAC-BoHV-4-AΔTK-KGK; 3, pBAC-BoHV-4-AΔTK-OvHV-2-gB. White arrows indicate expected differential bands generated by DNA digestion in each genome.

BAC homologous recombination using a galactokinase K (galK) and kanamycin selection systems was used to construct a recombinant BoHV-4 virus expressing the OvHV-2 gB (BoHV-4-AΔTK-OvHV-2-gB) (Figure 1). The BoHV-4 BAC carrying the galK and kanamycin resistance expression cassette (KGK) into the TK locus (pBAC-BoHV-4-AΔTK-KGK) used as a backbone genome was prepared as described elsewhere [11]. Genetic recombination and selection were performed according to previously described protocols [11,12]. Briefly, an OvHV-2 ORF8 cassette (CMV-OvgB-V5), containing a codon-optimized sequence of the OvHV-2 ORF 8 cloned downstream to the CMV promoter and in frame with the V5 epitope at the 3-prime end, was generated by PCR using pOvHV-2-ORF8 [6] as template and primers R1-OvgB and R2-OvgB (Table S1). The CMV-OvgB-V5 cassette was transformed into *E. coli* SW102 containing the pBAC-BoHV-4-AΔTK-KGK, to generate the pBAC-BoHV-4-AΔTK-OvHV-2-gB. Negative selection was performed on plates containing minimal medium and 2-deoxygalactose with glycerol as the carbon source, where only clones that had the *galK* gene replaced by the OvHV-2 ORF8 gene could grow. The selection was also confirmed by culturing selected clones in the presence of kanamycin, where clones containing the CMV-OvgB-V5 cassette would not grow.

DNA was extracted from BAC clones using NucleoBond^®^ BAC 100 (Clontech Laboratories, Inc., Mountain View, CA, USA) in accordance with the manufacturer’s recommendations. Correct insertion of the CMV-OvgB-V5 cassette was verified by PCR using primers P1–P4 (Table S1) flanking the recombination junctions between the CMV-OvgB-V5 cassette and the BoHV-4 TK gene (Figure 1A), followed by amplicon sequencing. The integrity of recombinant viruses was evaluated by examining their DNA restriction profile following *Eco*RI and *Hin*dIII digestions.

### 2.3. Reconstitution of Recombinant Viruses

The ability of pBAC-BoHV-4-AΔTK-OvHV-2-gB to reconstitute infectious viral particles was assessed by plaque formation in mammalian cells following BAC DNA transfection. Briefly, FMSK^htert.1^ cells grown to 70–80% confluency in 6-well plates were transfected with BAC DNA (1.2 μg/well of pBAC-BoHV-4-A, pBAC-BoHV-4-AΔTK-KGK or pBAC-BoHV-4-AΔTK-OvHV-2-gB) using Attractene Transfection Reagent (Qiagen, Valencia, CA, USA) in accordance with the manufacturer’s recommendations. Transfections were confirmed by the visualization of green fluorescence protein (GFP), expressed by the BAC cassette, using fluorescence microscopy. Virus reconstitution and spread were monitored by GFP expression and plaques formation. At five days post-transfection, the reconstituted virus was harvested after three freeze-thaws and clarified by centrifugation (3000× *g*, 30 min at 4 °C). The clarified virus supernatant was stored at −80 °C until used. 

### 2.4. OvHV-2 gB Expression

The expression of OvHV-2-gB by pBAC-BoHV-4-AΔTK-OvHV-2-gB was assessed by immunofluorescence and immunoblotting assays using transiently transfected HEK293T cells. Transfections were performed on 70–80% confluent cells in 6-well plates using Attractene Transfection Reagent (Qiagen, Valencia, CA, USA). DNA from pBAC-BoHV-4-A, pBAC-BoHV-4-AΔTK-KGK and pOvHV-2-ORF8/V5, which have been described elsewhere [6,11], were used as controls. At 24 h post-transfection, cells were washed 3 times in PBS, harvested using 10 mM EDTA, and processed for each assay.

For immunoblotting, the cells were lysed in 1X lysis buffer (Promega, Madison, WI, USA) and stored at −20 °C until used. An automated, capillary-based immunoassay system and a 12–230 kDa Wes separation module (Simple Western assays, Wes, ProteinSimple, San Jose, CA, USA) were used as per the manufacturer’s recommendations. Equivalent amounts of protein lysates were loaded in each well of the Wes assay plate. An anti-V5 (Invitrogen, Carlsbad, CA, USA) and the anti-OvHV-2 gB F1.2 monoclonal antibody, previously prepared in our laboratory (Supplemental Material), were used as primary antibodies. The reactions were detected by an HRP-conjugated anti-mouse antibody followed by chemiluminescence treatment. Images were obtained and analyzed using the Compass software v2.7 (ProteinSimple, San Jose, CA, USA). 

For immunofluorescence, cells were deposited onto wells of glass slides and immediately fixed (3:1 ratio of methanol and acetone) at −20 °C for 5 min. The cells were then probed with anti-OvHV-2 gB F1.2 or anti-V5 as primary antibodies and an Alexa Fluor^TM^ 594 goat anti-mouse IgG (H+L) as secondary antibody (Invitrogen, Carlsbad, CA, USA). Slides were mounted with Prolong Diamond Antifade Mountant with DAPI (Invitrogen) and examined using a fluorescence microscope. 

### 2.5. Viral Stocks and Titration 

Confluent monolayers of FMSK^htert.1^ or FMSK^htert.1^/Cre were infected with virus at a desired MOI in serum-free DMEM for 4–6 h. After that, cells were split, and medium was changed to c-DMEM. Cells were then cultured until CPE reached about 80% (3 to 4 days post-infection), and the virus was harvested by freezing and thawing cultures three times. Viral preparations were clarified by centrifugation and stored at −80 °C until use. 

To excise the loxP-flanked BAC cassette, FMSK^htert.1^/Cre cells were incubated with a virus suspension, cultured for 3 days, and transferred into uninfected FMSK^htert.1^/Cre cells. Following three passages, virus stocks were prepared as described above. The excision of the BAC cassette was monitored by loss of GFP expression in viral plaques using fluorescence microscopy.

The titer of virus stocks was determined as 50% tissue culture infectious dose (TCID_50_) following limiting dilution assay. Briefly, virus stock was serially diluted in c-DMEM and each viral dilution (100 μL/well) was added to 80–90% confluent FMSK^htert.1^ in 96-well plates and cultured for six days. Eight replicates of each viral dilution were used per assay. The presence or absence of plaques in each well was verified by microscopic examination following standard 10% formaldehyde fixation and 0.1% crystal violet staining. 

### 2.6. Viral Growth Curves

Triplicate cultures of FMSK^htert.1^ cells were infected with wild-type BoHV-4-A, BoHV-4-AΔTK-KGK, or BoHV-4-AΔTK-OvHV-2-gB at a MOI of 0.1. The cultures were incubated for 4 h at 37 °C and 5% CO_2_, and media was replaced by c-DMEM. At various time points post-infection, the cultures were transferred to −20 °C. Following two freeze-thaw cycles, infectious virus in each preparation was quantitated using the TCID_50_ method described above. 

### 2.7. Animal Trial

Fourteen 10-week-old New Zealand rabbits were obtained from Western Oregon Rabbit Co., Portland OR, and maintained at a Washington State University animal facility according to approved Federal and State regulations and protocols for animal welfare and use (WSU IACUC # 6364). The rabbits were randomly separated into two groups: OvHV-2 gB immunized (n = 7) and control (n = 7). The rabbits were monitored daily throughout the experiment. Blood samples were collected at specific intervals (vide infra) to evaluate viral load and immunological responses.

#### 2.7.1. Immunization Protocol

Recombinant BoHV-4-AΔTK-OvHV-2-gB, prepared in FMSK^htert.1^ cells, as described above, was used as the immunogen for the OvHV-2 gB immunized group. Supernatant from uninfected cells, prepared as the viral infected cells, was used as a mock immunogen in the control group. A prime immunization (day 0) was followed by two booster immunizations given at two-week intervals (days 14 and 28). All inoculations were delivered intravenously in a volume of 1 mL via marginal ear vein. For BoHV-4-AΔTK-OvHV-2-gB, a virus dose of 2 × 10^5^ TCID_50_ per immunization was used. 

#### 2.7.2. OvHV-2 Challenge

At 21 days post-last immunization, all 14 rabbits were challenged with a lethal dose (10^6^ OvHV-2 genome copies) of OvHV-2 delivered through nebulization. The viral inoculum consisted of nasal secretions from OvHV-2 infected-sheep that were prepared as previously described [13]. Following challenge, blood samples were collected at every other day to weekly intervals to monitor OvHV-2 infection and specific immune responses. The animals were examined daily for development of clinical signs. Any rabbit with a sustained rectal temperature above 40 °C was euthanized using a pentobarbital overdose within 48 h of the onset of fever. Healthy rabbits were euthanized at the end of the experiment at 64 days post-challenge. 

#### 2.7.3. Pathology

All animals were examined postmortem immediately after euthanasia. A duplicate set of samples from lung, liver, mesenteric lymph nodes, and spleen were collected. One set was frozen in liquid nitrogen for further molecular examination. The other set was collected into 10% neutral-buffered formalin. Following fixation, tissues were embedded in paraffin, sectioned 5-μm thick, and stained with hematoxylin and eosin (HE). Histological examination was performed by a pathologist and lesions were scored blindly. Tissues with no lesions were indicated as no visible lesion (NVL), mild lesions were scored as “+”; moderate, “++”; and severe “+++”. 

#### 2.7.4. Viral Detection

BoHV-4-AΔTK-OvHV-2-gB and OvHV-2 levels in blood and tissues were assessed by specific BoHV-4 and OvHV-2 quantitative PCR (qPCR) as described below. 

DNA from buffy coats and cryopreserved tissue samples was extracted and quantified using a QIAamp DNA Mini Kit (Qiagen, Valencia, CA, USA) and a fluorescence-based Qubit™ quantitation assay (Thermo Fisher Scientific, Hanover Park, IL, USA), respectively, as per the manufactures’ recommendations. 

The BoHV-4 qPCR targeted BoHV-4 ORF20 and used the inner primers of a previously published nested PCR [14,15] (Table S1). The PCR reaction (20 μL) contained 1X SsoFast Evagreen Supermix (BioRad, Irvine, CA, USA), 400 nM of each primer and template (20 ng of tested DNA or serial diluted plasmid DNA (amplicon inserted) used for standard curve). PCR reactions were cycled in a CFX thermocycler (BioRad) at 95 °C for 30 s followed by 40 cycles of 95 °C for 15 s, 56 °C for 15 s with a plate read after each cycle. A final melting curve was run from 60 °C to 90 °C with an increment of 0.5 °C every 5 s. The results were analyzed on the CFX Manager software and reported as BoHV-4 genome copies per 20 ng of total DNA.

A qPCR targeting the OvHV-2 ORF 75 [16,17] was used to quantify viral load after challenge. The qPCR was performed as described by Traul et al., 2007 [17] using primers listed on Table S1, with the exception that a CFX thermocycler and CFX Manager software were used. The results were reported as OvHV-2 genome copies per 50 ng of total DNA.

#### 2.7.5. BoHV-4 and OvHV-2 gB ELISA

A commercial BoHV-4 ELISA (BIO-X, Brussels, Belgium) was used to assess anti-BoHV-4 antibodies. The assays were performed per the manufacturer’s instructions and results shown as the ratio between the OD of a tested plasma by the average OD of the negative control, both tested at 1:100 dilution. 

For the detection of antibodies specific to OvHV-2 gB, an ELISA based on recombinant OvHV-2 gB was used as previously described [6] with slight modifications. Briefly, protein extracts prepared from HEK293T cells transfected with the OvHV-2-gB plasmid and diluted at 1:500 were used as antigen and rabbit plasma samples were tested at 1:400 dilution. Following standard antibody binding, washes, and detection, ELISA results were reported as a ratio of the tested sample OD divided by the background OD.

#### 2.7.6. Viral Neutralization Assay

To assess the neutralizing activity of OvHV-2 antibodies, an assay based on a recombinant alcelaphine herpesvirus 1 (AlHV-1) whose gB was replaced by the OvHV-2 gB was used as previously described [18]. Briefly, serially diluted plasma (1:8 to 1:256) was mixed with 10^2^ TCID_50_ rAlHV-1/OvHV-2-gB and incubated for 1 h at 37 °C. The plasma and virus mixture (four replicates for each plasma dilution) was added to FMSK^htert.1^ cells (2.5 × 10^4^ cells/well of a 96-well plate) and cultured for six days. Assay controls included in each plate comprised of uninfected cells, cells incubated with untreated virus, and virus treated with standard positive plasma. Following the final incubation step, the cells were fixed and stained as described for TCID_50_, and the presence or absence of plaques was recorded. Virus neutralizing activity against OvHV-2 gB was calculated as neutralization titer expressed as the reciprocal of the highest plasma dilution that resulted in no plaques in at least one of the replicates.

### 2.8. Data Analyses

All graphical images and statistical analyses were performed on GraphPad Prism 9.0.0 for Windows (GraphPad Software, San Diego, CA, USA, www.graphpad.com). Virus growth was compared by one-way ANOVA with the Geisser-Greenhouse correction. Fisher Exact Test was used to compare protection rates in the vaccine trial, while time for development of clinical signs, viral load in tissues and antibody responses in the OvHV-2 immunized and control groups were compared using Mann–Whitney test. *p*-values equal or lower than 0.05 were considered statistically significant for all analyses.

## 3. Results

### 3.1. Recombinant BoHV-4 Construction and In Vitro Characterization

BAC recombineering using *E. coli* SW102 successfully replaced the KGK gene cassette in the pBAC-BoHV-4-AΔTK-KGK with the CMV-OvgB-V5 cassette resulting in construction of pBAC-BoHV-4-AΔTK-OvHV-2-gB (Figure 1). Correct recombination events were confirmed using PCR, sequencing and restriction enzyme digestions. PCR resulted in the amplification of expected band sizes using the junction PCR (spanning recombination sites between the CMV-OvgB-V5 cassette and the BoHV-4 TK gene). The sequencing of amplicons matched with the expected sequences of the inserted cassettes and their location into the BoHV-4 TK locus. DNA digestions resulted in the expected restriction patterns for each of the recombinant viruses using *Eco*RI and *Hin*dIII (Figure 1B), confirming the overall integrity of their genomes following recombination events. 

**Figure 2:**
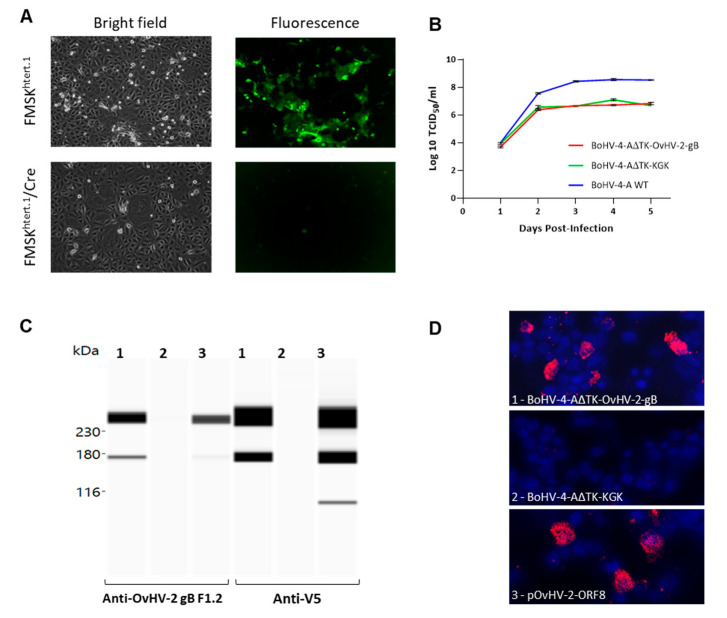
BoHV-4-A∆TK-OvHV-2-gB infectivity and OvHV-2 gB expression. (**A**) Representative microscopic images of plaques formed by virus reconstituted from pBAC-BoHV-4-A∆TK-OvHV-2-gB in FMSK^htert.1^ and FMSK^htert.1^/Cre cells. (**B**) Replication kinetics of BoHV-4-A∆TK-OvHV-2-gB, BoHV-4-A∆TK-KGK, and BoHV-4-A in FMSK^htert.1^ cells. Growth curves show mean TCID50/mL data from triplicate measurements. Error bars are SEM. Expression of OvHV-2 gB was examined in HEK293T cells transfected with (#1) BoHV-4-A∆TK-OvHV-2-gB, (#2) BoHV-4-AΔTK-KGK, or (#3) pOvHV-2-ORF8 (positive control) by immunoblotting (**C**) and immunofluorescence (**D**). For immunoblotting, reaction with both anti-OvHV-2 (F1.2) and anti-V5 antibodies are shown (**C**). For immunofluorescence, reaction with the anti-OvHV-2 (F1.2) is shown in representative confocal images (20✕); OvHV-2 gB is stained in red (Alexa Fluor^TM^ 594 goat anti-mouse IgG (H+L), used as secondary antibody) and cell nuclei in blue (DAPI).

To evaluate the viability of the virus reconstituted from the new generated BAC, pBAC-BoHV-4-AΔTK-OvHV-2-gB DNA was transfected into FMSK^htert.1^ and the cells cultured for five days. Green plaques were visualized, confirming the presence of infectious viruses expressing GFP encoded by the BAC cassette (Figure 2A, FMSK^htert.1^). The reconstituted virus used to infect FMSK^htert.1^/Cre also generated plaques but green fluorescence was lost after three cell passages indicating excision of the BAC cassette (Figure 2A, FMSK^htert.1^/Cre). The ability of the reconstituted virus to replicate was also confirmed by the increasing titer observed at 2- and 3-days post-infection when growth curves were evaluated. The virus reconstituted from pBAC-BoHV-4-A∆TK-OvHV-2-gB grew at a rate comparable to the one reconstituted from pBAC-BoHV-4-A∆TK-KGK, but with lower replication kinetics than the pBAC-wild-type BoHV-4-A (*p* = 0.26 and *p* = 0.02, respectively), as shown by approximately 2 log units lower titers starting at 3 days post-infection (Figure 2B). OvHV-2 gB expression by BoHV-4-A∆TK-OvHV-2-gB was confirmed by immunoblotting and immunofluorescence using both an anti-OvHV-2 gB, F1.2, and an anti-V5 monoclonal antibodies. Immunoblotting bands comparable to the control, protein lysates from cells transfected with a plasmid expressing OvHV-2 gB, were also observed in cells transfected with BoHV-4-A∆TK-OvHV-2-gB but not with BoHV-4-A∆TK-KGK (Figure 2C). Similar results were obtained by immunofluorescence, reactivity using both primary antibodies were detected in cells transfected with either the BoHV-4-A∆TK-OvHV-2-gB or the plasmid control (expressing OvHV-2 gB) but not with BoHV-4-A∆TK-KGK (Figure 2D, representative images of F1.2 monoclonal antibody reactivity). 

### 3.2. Vaccine Trial in a Rabbit Model

**Figure 3:**
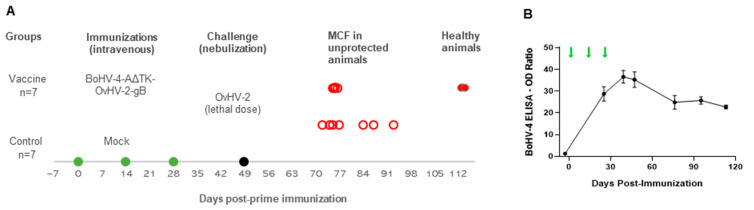
Vaccine-challenge trial in a rabbit model. (**A**) Experimental design and disease outcome upon challenge. Two groups of seven rabbits each were immunized with either BoHV-4-A∆TK-OvHV-2-gB or with a mock preparation. Green dots indicate immunizations, the black solid dot indicates challenge, and red circles show the number of animals that developed malignant catarrhal fever (MCF) (open) or remained healthy (solid) until the end of the experiment at 113 days post-prime immunization. (**B**) BoHV-4 antibody response in rabbits vaccinated with BoHV-4-A∆TK-OvHV-2-gB, showing mean ELISA OD ratio and SEM. Green arrows indicate immunization times.

The safety and efficacy of the newly constructed virus, BoHV-4-A∆TK-OvHV-2-gB, was tested by an immunization-challenge trial using rabbits as a model. Figure 3A shows a schematic representation of events in the trial, including immunizations, challenge, the development of MCF in unprotected animals, and experiment termination. Importantly, no adverse effect from immunizations was observed in any of the experimental animals and all 14 rabbits were healthy at the time of challenge. 

To monitor viral infection following immunizations, BoHV-4 PCR was performed on blood samples and in splenic samples collected at necropsy. BoHV-4 DNA was not detected in blood or tissues at any time point tested. Despite the absence of detectable BoHV-4 DNA in blood, all animals vaccinated with BoHV-4-A∆TK-OvHV-2-gB seroconverted following prime and booster immunizations and showed a sustained BoHV-4-specific antibody response throughout the study, as detected by ELISA (Figure 3B). This indicates that immunizations resulted in BoHV-4 infection without a detectable viremia or latency. 

At 21 days after the last immunization, all 14 rabbits were challenged with a minimal lethal dose of OvHV-2 by intranasal nebulization to evaluate the protective effects of the vaccine against SA-MCF. Infection with OvHV-2 resulted in SA-MCF in all seven mock-vaccinated rabbits. Four OvHV-2-gB vaccinated rabbits also developed SA-MCF, while the remaining three vaccinated rabbits remained healthy until the end of the experiment at 64 days post-challenge (Figure 3A). Although vaccination was associated with survival of three of seven rabbits (Table 1), the proportion of protected (42.9%) and unprotected (57.1%) animals in the vaccinated group did not show a statistically significant difference in comparison with controls (*p* = 0.19).

**Figure 4:**
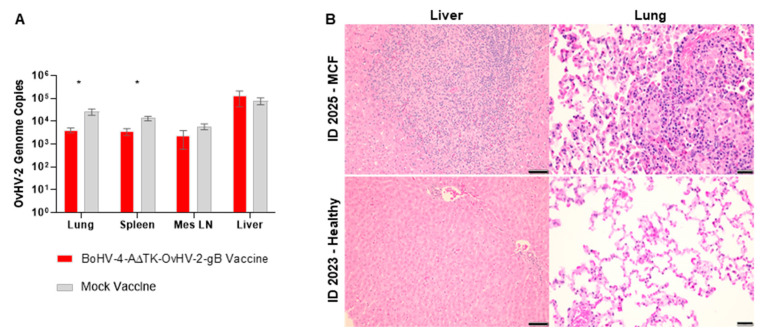
Development of MCF following immunization with BoHV-4-A∆TK-OvHV-2-gB and challenge with OvHV-2 in a rabbit model. (**A**) OvHV-2 genome copies in tissues of BoHV-4-A∆TK-OvHV-2-gB and mock immunized rabbits. Results are mean OvHV-2 genome copies per 50 ng of total tissue DNA quantified by OvHV-2 qPCR and error bars represent SEM. *, indicates a *p* < 0.05 as tested by Mann–Whitney test. (**B**) Representative histological images of liver and lung of BoHV-4-A∆TK-OvHV-2-gB vaccinated rabbits that developed MCF (ID 3025) or remained healthy (ID 3023) following OvHV-2 challenge. Rabbit ID 3025: liver, moderate portal and periportal hepatitis with portal bridging (bar: 50 µm); lung, mild focal pneumonia with a predominance of macrophages and small lymphocytes (bar: 20 µm). Rabbit ID 3023: unremarkable liver (bar: 50 µm); unremarkable lung (bar: 20 µm).

Following the challenge, OvHV-2 infection and viral load were assessed by qPCR in blood and tissue samples, while the development of MCF was confirmed by histological examination. The three rabbits that were protected from disease following challenge showed no clinical signs of OvHV-2 infection; no OvHV-2 DNA was detected in their blood throughout the experiment, nor in tissues after euthanasia; and no histological changes typical of MCF developed. All rabbits that developed MCF following challenge had a similar clinical course, regardless of their immunization status. These unprotected animals developed fever and were euthanized at 24–48 h onset of temperature ≥40 °C. The time from first detection of OvHV-2 DNA in blood and euthanasia was similar in both groups, 7.5 ± 0.5 days for the BoHV-4-A∆TK-OvHV-2-gB vaccinated and 8.14 ± 2.3 days for the control group (*p* = 0.88). Overall, there were lower viral loads in tissues of the vaccinated group compared to the non-vaccinated (Figure 4A), although statistically significant differences were detected only in the lung and spleen (lung, *p* = 0.04; spleen, *p* = 0.02; mesenteric lymph node, *p* = 0.23; liver, *p* = 0.78). All rabbits that developed disease had lesions typical of MCF [13]. The pathological changes and lesion severity scores are summarized in Table 2. The most consistent change was moderate to severe disseminated portal-periportal hepatitis, accompanied by portal-portal bridging. Many larger hepatic lesions were grossly evident at necropsy, manifested as focal or multifocal areas of white discoloration (1–5 mm). Infiltrates consisted of epithelioid macrophages mixed with small lymphocytes, and scant heterophils and plasma cells. There was mild bile duct hyperplasia. Pulmonary lesions were generally mild, particularly in the vaccinated rabbits. The small perivascular inflammatory foci (230–260 µm) were typically adjacent to small bronchioles and had the same cellular composition as those in liver. Mild histiocytic alveolitis was present at the margins of foci. Moderate lymphoid hyperplasia was observed in mesenteric lymph nodes of two BoHV-4-A∆TK-OvHV-2-gB vaccinated rabbits. No vasculitis developed in any of the tissues examined. Illustrated in Figure 4B is a comparison of changes in lung and liver in a BoHV-4-A∆TK-OvHV-2-gB vaccinated rabbit that developed MCF (ID 3025) with a rabbit in the same group that responded to vaccination and was free of lesions at the end of the study (ID 3023). 

**Figure 5:**
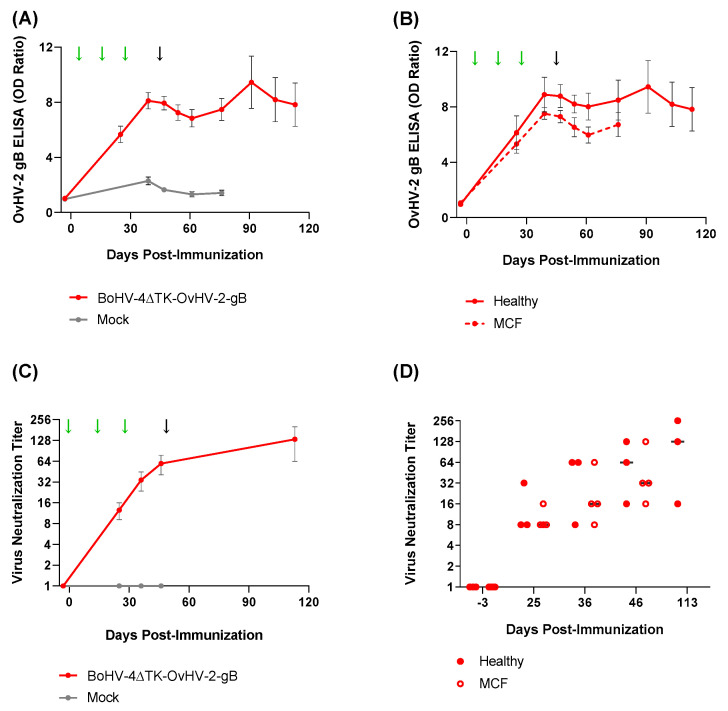
Antibody response induced by immunization with BoHV-4-A∆TK-OvHV-2-gB. Anti-OvHV-2 gB IgG (H+L) detected by ELISA in plasma samples from OvHV-2 gB vaccinated and control groups (**A**) and in OvHV-2-gB vaccinated animals that were protected (healthy) or unprotected (MCF) following OvHV-2 challenge (**B**). OvHV-2 gB neutralizing antibody activity in OvHV-2-gB- and mock-immunized animals (**C**) and individual neutralization titer (median) of vaccinated animals that were protected (healthy) following OvHV-2 challenge or unprotected and developed MCF (**D**). Error bars are SEM and arrows indicate days when animals were immunized (green) and challenged (black).

To evaluate antibody responses elicited to OvHV-2 gB by vaccination and the subsequent OvHV-2 challenge, plasma samples were tested by both ELISA and viral neutralization assays (Figure 5). Immunization with BoHV-4-A∆TK-OvHV-2-gB resulted in increased levels of anti-OvHV-2 gB antibodies in blood, which peaked on average after the third immunization and were sustained throughout the experiment. The slight peak observed at 42 days post-OvHV-2 challenge, shown in Figure 5A,B, was seen because of an increased response in only one of the three animals. No specific antibody response was observed in the control group; with the exception of the pre-immunization time-point, when all animals were seronegative to OvHV-2 gB, significantly higher antibody levels were detected in the OvHV-2 gB vaccinated group at all time points tested after vaccination or challenge as compared to control (*p* < 0.001 at all time-points) (Figure 5A). No difference in systemic antibody response to OvHV-2 gB was observed between vaccinated animals that remained healthy and the vaccinated animals that developed MCF following OvHV-2 challenge (*p* > 0.05 at all time-points) (Figure 5B). 

Importantly, antibodies from plasma of BoHV-4-A∆TK-OvHV-2-gB-immunized animals blocked rAlHV-1/OvHV-2-gB in a neutralization assay. No neutralization effect was observed with plasma collected prior to immunization or from mock-vaccinated animals after immunization or challenge (Figure 5C). Neutralizing antibodies were induced by vaccination with OvHV-2 gB and neutralization titers ranging from 16 to 128 were detected prior to OvHV-2 challenge (Table 2). The three rabbits that survived the challenge had neutralization titers of 16, 128 and 256 at the end of the study (64 days post-challenge) (*p* > 0.05 at pre immunization, and *p* = 0.002 at all other time-points). Among OvHV-2 gB vaccinated rabbits, the ones that were protected from MCF following challenge tended to have higher virus neutralization titers than the ones that succumbed to disease; this difference could not be confirmed statistically (*p* > 0.05).

## 4. Discussion

In this study we tested whether immunization with OvHV-2 gB, delivered by a BoHV-4-vector, induced antibody response and protection against SA-MCF. For that, a new recombinant BoHV-4 virus containing the full-length OvHV-2 ORF 8, encoding gB, was constructed using a recombination system based on galK selection. The in vitro characterization of the newly constructed virus showed expected recombination events that resulted in correct insertion of the OvHV-2 gB gene in the BoHV-4 TK locus. While the deletion of TK gene can affect viral fitness [19], as observed by slower replication kinetics when compared to wild-type BoHV-4, it did not affect the ability of the recombinant virus to infect and replicate in permissive cells. BoHV-4-A∆TK-OvHV-2-gB, was infectious, grew in cell culture at a similar kinetic rate as its parental virus and expressed OvHV-2 gB in mammalian cells in vitro. These results prompted us to test this new virus as a vector to deliver OvHV-2 gB as a vaccine to SA-MCF, using a rabbit model.

The rabbits were immunized with BoHV-4-A∆TK-OvHV-2-gB delivered by intravenous inoculation. This approach was used previously to evaluate immunogenicity of heterologous antigens delivered by BoHV-4 [11,19]. As expected, based on previous studies using rabbits as a model for BoHV-4 infection, BoHV-4-A∆TK-OvHV-2-gB was safe and no adverse effects developed in any animal. Although BoHV-4 is considered by some to be a pathogen in cattle [20,21], its natural host, such effects have not been confirmed or demonstrated experimentally [22,23,24]. This suggests that its pathogenicity may be secondary to other agents. Therefore, BoHV-4 continues to be a valuable system to deliver antigens as vaccines.

All rabbits inoculated with BoHV-4-A∆TK-OvHV-2-gB in this study seroconverted to BoHV-4 antibodies, confirming infection. However, the persistence of the latent virus could not be confirmed. Although persistence of wild-type BoHV-4 following experimental infection in rabbits has been reported [25,26], the fate of recombinant BoHV-4 tested as vaccine vectors in different species remains uncertain. The viral genome modifications performed to obtain the BoHV-4-A∆TK-OvHV-2-gB recombinant virus are unlikely to affect viral latency, but further analysis remains to be done to confirm viral distribution upon infection and persistence. If the recombinant virus did establish latency in the immunized rabbits based on the sustained immune response to BoHV-4, a plausible explanation for the lack of detection of BoHV-4 DNA in blood or tissues is that viral load in the samples tested was below the assay threshold. Similar results were obtained in another study where a recombinant BoHV-4, expressing selected BVDV and BoHV-1 proteins encoded by genes inserted in the BoHV-4 TK locus, was tested in cattle [9]. In that study, BoHV-4 DNA was not detected in tissues and attempts to recover virus in cell culture were unsuccessful. Nevertheless, an increased immune response to the heterologous proteins was observed following dexamethasone treatment, suggesting reactivation of the viral vector with re-stimulation of the immune system. For the standpoint of vaccination, viral persistence is an advantage as it may continue to express the target antigen and boost the immune response. Therefore, the ability of recombinant BoHV-4 to persist is an important consideration and requires further investigation.

In this study, challenge with OvHV-2 resulted in infection and development of SA-MCF in all seven animals in the control group, while three of seven (42.9%) rabbits immunized with BoHV-4-A∆TK-OvHV-2-gB remained healthy after exposure to a lethal dose of OvHV-2. This suggests that the viral construction has potential as a vaccine for SA-MCF, although modifications are necessary to improve efficacy. 

The sub-optimal protection provided by BoHV-4-A∆TK-OvHV-2-gB may be due to low levels of expression of OvHV-2 gB in vivo. Besides OvHV-2 gB, the recombinant virus also expresses the BoHV-4 gB; therefore, the OvHV-2 protein is not essential for the virus and its expression may be reduced following infection. Replacement of BoHV-4 gB by the OvHV-2 counterpart may result in higher expression levels by the recombinant virus. This possibility is currently being evaluated. Another opportunity to increase efficacy of the vaccine would be to incorporate additional OvHV-2 glycoproteins in the recombinant virus, such as gH and gL. As previously demonstrated to OvHV-2 gB, antibodies specific to OvHV-2 gH and gL can also block OvHV-2 entry into cells and are potential vaccine candidates [6]. Alternatively, adjuvants may also be used to increase vaccine efficacy. It has been shown with the attenuated AlHV-1 vaccine for wildebeest-associated-MCF that delivery of the vaccine in Emulsigen^®^ (MVP Adjuvants) is critical to induce protective antibody responses [27,28,29]. 

The role of antibodies induced by vaccination in protection against MCF have been demonstrated in previous studies using an attenuated AlHV-1 vaccine [27,28,29,30,31]. Although results vary depending on animal breed, the adjuvant used, and the experimental design, overall, high levels of vaccination-induced specific antibodies in the respiratory tract and blood are associated with protection following challenge. In our study, all rabbits immunized with BoHV-4-A∆TK-OvHV-2-gB developed a specific humoral antibody response with demonstrated neutralizing activity, indicating that the antigen delivered by the recombinant BoHV-4 was immunogenic. Overall, antibody levels and neutralizing activity were lower in animals that developed MCF than the ones that were protected from disease, although no statistically significant difference was observed, possibly due to high individual variability. Despite this, it is likely that increased levels of antibodies contributed, at least in part, to protection. Interestingly, rabbit 3025, which had one of the highest neutralizing titers prior to OvHV-2 challenge, was not protected, suggesting that systemic anti-OvHV-2 gB neutralizing antibodies alone are not protective. While not the focus of this study, other immune responses such as mucosal and cellular immune responses may also play important roles in protection against SA-MCF and need further investigation. 

## 5. Conclusions

In this study, we demonstrate that immunization with OvHV-2 gB delivered by a recombinant BoHV-4 can induce humoral immunity against SA-MCF in a rabbit model. Despite the antibody response induced by immunization with BoHV-4-A∆TK-OvHV-2-gB, only a moderate protection rate was observed following OvHV-2 challenge. Improvements such as the inclusion of additional viral targets or use of adjuvants are still needed to increase vaccine efficacy. Nevertheless, this study represents an important step towards the development of an effective SA-MCF vaccine. 

## Figures and Tables

**Table 1:** Protection rate of a BoHV-4-A∆TK-OvHV-2-gB vaccine following OvHV-2 lethal challenge in a rabbit model.

	Vaccine		Total
	BoHV-4-A∆TK-OvHV-2-gB	Mock	
Protected	3 (42.9%)	0 (0%)	3
Unprotected (MCF)	4 (57.1%)	7 (100%)	11
Total	7	7	14

**Table 2:** Health status, neutralizing antibody response, and lesion scores in an SA-MCF vaccine-challenge trial using a recombinant BoHV-4 delivering OvHV-2 gB in a rabbit model.

Vaccine ^1^	Challenge ^2^	Animal ID	Neutralizing Antidodies ^3^	DPC	Health Status	Lesion Scores ^4^			
						Lung	Liver	Spleen	Mes LN
BoHV-4-A∆TK- OvHV-2gB	OvHV-2	3020	pos (16)	64	Healthy	NVL	NVL	NVL	NVL
		3021	pos (32)	27	MCF	NA	++	NVL	++
		3022	pos (16)	26	MCF	+	++	NVL	++
		3023	pos (128)	64	Healthy	NVL	NVL	NVL	NVL
		3024	pos (32)	26	MCF	+	++	NVL	NVL
		3025	pos (128)	27	MCF	+	+++	NVL	NVL
		3026	pos (64)	64	Healthy	NVL	NVL	NVL	NVL
Mock	OvHV-2	3030	Neg	26	MCF	+	+++	NVL	NVL
		3031	Neg	38	MCF	+	+	NVL	NVL
		3032	Neg	35	MCF	++	++	NVL	NVL
		3033	Neg	25	MCF	+	++	NVL	NVL
		3034	Neg	28	MCF	+	++	NVL	NVL
		3035	Neg	44	MCF	+	+	NVL	NVL
		3036	Neg	23	MCF	++	+++	NVL	NVL

DPC, days post-challenge when animal was euthanized; Mes LN, mesenteric lymph node; ^1^, rabbits vaccinated with the BoHV-4-vectored OvHV-2 gB (BoHV-4-A∆TK OvHV-2 gB, 2 × 10^5^ TCID_50_ per immunization) or culture medium only (mock). Three intravenous immunizations at 2-week intervals; ^2^, rabbits challenged with OvHV-2 (10^6^ viral DNA copies) by intranasal nebulization at 49 days post-prime immunization; ^3^, Presence (pos) or absence (neg) of OvHV-2 gB-specific neutralizing antibodies at 46 days post-prime immunization (3 days before challenge); antibody titer is indicated in parenthesis; ^4^, NVL, no visible lesion. Lesion score: +, mild; ++, moderate; and +++, severe lesions; NA, tissue not available.

## Data Availability

The data presented in this study are available on request from the corresponding author.

## Supplementary Materials

The following are available online at https://www.mdpi.com/2076-393X/9/2/90/s1, Table S1. Primers used to create the CMV-OvHV-2-gB-V5 cassette and confirm its correct insertion into pBAC-BoHV-4-AΔTK-OvHV-2-gB.
