# Supplementary material for: OvHV-2 Glycoprotein B Delivered by a Recombinant BoHV-4 Is Immunogenic and Induces Partial Protection against Sheep-Associated Malignant Catarrhal Fever in a Rabbit Model
Source: Vaccines (Basel). 2021 Jan 26;9(2):90. doi: 10.3390/vaccines9020090 (PMC7911203; doi:10.3390/vaccines9020090)
Supplement: Supplementary file 1 [file vaccines-09-00090-s001.pdf]

Table S1. PCR primers and probes used in the study.

Primer	5′-3′ sequences
**R1**-OvgB	**cagtcttttcggactgtcataaacttatttatgatgtcatgaagatgggaaaacatggcagttttagt gtctcctcaaagctt**gttgacattgattattg
**R2**-OvgB	**aacatcactaatgggaaaaccaaacatgcagataaggcatgtctatcaaacatgacccagtgact gacatccgaacggccc**ggataccccctagagcccc
P1 (P67co-LF2.1)	ggcacacttgttataccccagacca
P2(Ov-optORF8-185 5' R)	agccctcctcctcattcag
P3(Ov-optORF8-163 3' F)	ccaccagaccaagcaggata
P4(LargeTK R2)	ccacgtattgtaaactgcaac
BoHV-4 ORF20 F	ttgatagtgcgttgttgggatgtgg
BoHV-4 ORF20 R	cactgcccggtgggaaatagca
OvHV-2-F	tggtaggagcaggctaccgt
OvHV-2-R	atcatgctgacccccttgcag
OvHV-2-P	tccacgccgtccgcactgtaaga

R1-OvgB and R2-OvgB were used to create the CMV-OvHV-2-gB-V5 cassette R1 and R2 sequences, homologous to the TK locus, are in bold letters.

P1-P4 were used to confirm correct insertion of the CMV-OvHV-2-gB-V5 cassette into pBAC-BoHV-4-AΔTK-OvHV-2-gB. Primer's references: P1; P2; P3; P4.

BoHV-4 ORF20 F and BoHV-4 ORF20 F used for amplification of BoHV-4 DNA.

OvHV-2-F, OvHV-2-R and OvHV-2-P were used for amplification and detection of OvHV-2 DNA.

Anti-OvHV-2 gB monoclonal antibody. One of the monoclonal antibodies used to detect OvHV-2 gB expression, F1.2, was prepared in our laboratory. The antibody was produced in mice following a series of immunizations using a biolistic system to deliver a plasmid expressing the OvHV-2 ORF 8 intradermally. The plasmid used for immunizations, pOvHV-2-ORF8, has been previously described (Cunha et al., 2015). The plasmid consists of a codon-optimized OvHV-2 ORF8 cloned into pCDNA3.2/V5 (Invitrogen); OvHV-2 ORF 8 is expressed under control of a CMV promoter as an OvHV-2 gB fused to the V5 epitope. Spleen of hyperimmunized mice were collected and processed by standard methods for production of monoclonal antibodies. Antibodies showing specific reactivity to OvHV-2 gB were screened and selected by ELISA and immunoblotting.
